# Long-Read Sequencing Identified a Large Novel *δ*/*β*-Globin Gene Deletion in a Chinese Family

**DOI:** 10.1155/2023/2766625

**Published:** 2023-10-04

**Authors:** Jianlong Zhuang, Yu Zheng, Yuying Jiang, Junyu Wang, Shuhong Zeng, Nansong Liu

**Affiliations:** ^1^Prenatal Diagnosis Center, Quanzhou Women's and Children's Hospital, Quanzhou, 362000 Fujian, China; ^2^Yaneng BIOscience (Shenzhen) Co., Ltd., Shenzhen, 518000 Guangdong, China

## Abstract

**Objective:**

Increasingly rare thalassemia has been identified with the advanced use of long-read sequencing based on long-read technology. Here, we aim to present a novel *δ*/*β*-globin gene deletion identified by long-read sequencing technology.

**Methods:**

Enrolled in this study was a family from the Quanzhou region of Southeast China. Routine blood analysis and hemoglobin (Hb) capillary electrophoresis were used for hematological screening. Genetic testing for common *α-* and *β*-thalassemia was carried out using the reverse dot blot hybridization technique. Long-read sequencing was performed to detect rare globin gene variants. Specific gap-polymerase chain reaction (gap-PCR) and/or Sanger sequencing were further used to verify the detected variants.

**Results:**

None of the common *α-* and *β*-thalassemia mutations or deletions were observed in the family. However, decreased levels of MCV, MCH, and abnormal Hb bands were observed in the family members, who were suspected as rare thalassemia carriers. Further, long-read sequencing demonstrated a large novel 7.414 kb deletion NG_000007.3:g.63511_70924del partially cover *HBB* and *HBD* globin genes causing delta-beta fusion gene in the proband. Parental verification indicated that the deletion was inherited from the proband's father, while none of the globin gene variants were observed in the proband's mother. In addition, the novel *δ*/*β*-globin gene deletion was further verified by gap-PCR and Sanger sequencing.

**Conclusion:**

In this study, we first present a large novel *δ*/*β*-globin gene deletion in a Chinese family using long-read sequencing, which may cause *δβ*-thalassemia. This study further enhances that long-read sequencing would be applied as a sharp tool for detecting rare and novel globin gene variants.

## 1. Introduction

It is well known that thalassemia is an inherited hemolytic anemia caused by the reduced or absent synthesis of one or more globin chains in hemoglobin due to globin gene deletions or mutations [1–3]. In China, *α*- and *β*-thalassemia were the most common genotypes; among them, most *α*-thalassemia was caused by a deletion in the *α*-globin gene, while *β*-thalassemia was mainly caused by point mutations in the *β*-globin gene [4, 5]. A high prevalence of thalassemia was observed in southern China, including Guangdong, Guangxi, Fujian, and other neighboring provinces [6–10]. Quanzhou region, located along the southeastern coastal regions of China, has a large population and high population mobility, as such, rare and novel thalassemia and hemoglobinopathy have been increasingly identified [11–13].

Traditional molecular diagnosis of thalassemia is usually based on PCR reverse dot blot hybridization (PCR-RDB) and/or gap-PCR [14] but all these methods have limitations in the identification of rare or novel thalassemia. Presently, long-read sequencing characterized with long reads has been gradually used in the molecular diagnosis of thalassemia and hemoglobinopathy. Long-read sequencing has an obvious advantage in identifying single-nucleotide variants, indels, and structural variants [15, 16]. In recent years, more and more rare and novel globin gene variants that cause thalassemia and hemoglobinopathy have been identified using long-read sequencing [15, 17–19]. In addition, a previous study recommended that long-read sequencing would be clinically utilized as an effective thalassemia carrier screening approach for at-risk couples [20].

In this study, long-read sequencing was performed to screen globin gene variants in a Chinese family with abnormal hematological results. A novel large 7.414 kb deletion NG_000007.3:g.63511_70924del partially cover *HBB* and *HBD* globin genes causing delta-beta fusion gene was identified in the family. This finding would strengthen the advantages of long-read sequencing in the molecular diagnosis of rare and novel thalassemia and provide more reference for optimizing the prevention and control of thalassemia.

## 2. Materials and Methods

### 2.1. Subjects

Enrolled in this study was a Chinese family from the Quanzhou region of Fujian province, Southeast China. All the subjects of this study deny receiving blood transfusions recently. After clinical consultation and signed informed consent, peripheral blood samples from this family were collected for further genetic analysis. This study was approved by the ethics committee of The Women's and Children's Hospital of Quanzhou (2021No.61).

### 2.2. Hematological Screening

Routine blood analysis was conducted on an automated cell counter (Sysmex XS-1000i; Sysmex Co., Ltd., Kobe, Japan) for mean corpuscular volume (MCV) and mean corpuscular hemoglobin (MCH) level detection. Hb capillary electrophoresis (Sebia, Evry Cedex, France) was carried out for Hb A, Hb A2, Hb F, and other abnormal Hb level detections. Positive thalassemia screening results were indicated as a MCV < 82 fL and/or a MCH < 27 pg and/or Hb A2 > 3.4% or Hb A2 < 2.6% or Hb F > 2.0%, or abnormal Hb bands.

### 2.3. Common Thalassemia Gene Testing

Subjects with abnormal hematological screening results were subject to thalassemia gene testing. The genomic DNA of the subjects in the family was extracted using an automatic nucleic acid extractor (Ruibao Biological Co., Ltd.). The PCR reverse dot hybridization technique (PCR-RDB) was used to detect the 23 common *α*-thalassemia and *β*-thalassemia variants in Chinese populations (Yaneng Biological technology Co., Ltd., Shenzhen) according to the manufacturer's protocol [21].

### 2.4. Long-Read Sequencing and Data Analysis

The genomic DNA of the enrolled family was obtained and then sent to an independent laboratory (Berry Genomics, Beijing) for long-read sequencing based on the PacBio Sequel II platform. The long-read sequencing for thalassemia detection was conducted according to our previously described manufacturing protocols [17]. Firstly, the purified DNA samples were quantified using the Qubit dsDNA BR assay kit (Thermo Fisher Scientific). Then, optimized primers were used to generate specific amplicons that encapsulate known structural variation regions and single nucleotide variation in the *HBA1*/*2* and *HBB* globin genes according to databases of HbVar, Ithanet, LOVD, and LOVD-China. After purification and end repair, double barcode adapters were ligated to the 5′ and 3′ ends, and Sequel Binding and Internal Ctrl Kit 3.0 (PacBio) was used to prepare SMRT bell libraries. Long-read sequencing was performed on the PacBio Sequel II System after primed DNA-polymerase complexes were loaded onto SMRT cells (PacBio).

Following the alignment of the subreads, the consensus circular sequence was mapped to the GRCh38 reference and variants called FreeBayes software, version 1.2.0. Linkage analysis (in cis or trans) in the long-read-based phasing was conducted using WhatsHap (version 0.18) software. Alignments of variant and wild-type molecules were manifested by the Integrative Genomics Viewer. Specific gap-PCR and Sanger sequencing were used to confirm the rare or novel globin gene deletions. In addition, rare globin gene sequence variants were verified by Sanger sequencing.

### 2.5. Specific Gap-PCR Amplification and Sanger Sequencing

Gap-PCR was used to identify the deletion breakpoints. We designed specific primers according to the known DNA sequences around the breakpoints. These primer sequences were P1: AGAGATGCGGTGGGGAGATA and P2: AACGATCCTGAGACTTCCACA. All primers were synthesized at Sangon Biotech (Shanghai). Gap-PCR reaction system: 5 × buffer 5 *μ*L, 25 mmol dNTPs 0.2 *μ*L, 25 mmol MgCl_2_ 1.5 *μ*L, Taq enzyme 2.5 U, 10 *μ*mol primers 1 *μ*L each, template 2 *μ*L, and plus ultrapure water to 25 *μ*L. The amplification conditions were 95°C for 10 min, then 35 cycles of 94°C for 1 min, 62°C for 30 s, 72°C for 1 min, and finally 72°C for 5 min. Electrophoresis analysis was performed, and the purified electrophoresis products were then sent for Sanger sequencing. The sequenced data were analyzed with GenBank NG_000007.3 as their reference sequences.

## 3. Results

### 3.1. Hematological Screening Results

**Figure 1:**
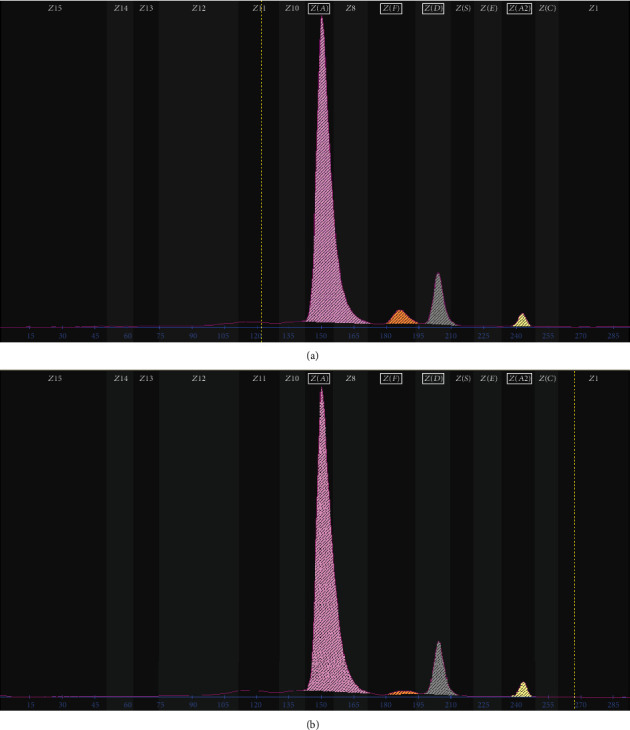
The results of hemoglobin capillary electrophoresis analysis in the family. (a) Hb capillary electrophoresis results elicited a decreased level of Hb A2 (2.3%), an increased level of Hb F (3.8%), and an abnormal Hb band in Hb zone 6 (9.8%) in the proband. (b) A similar Hb capillary electrophoresis result was also observed in the proband's father.

The hematological screening results of the enrolled family are listed in Table 1. Routine blood analysis elicited decreased levels of MCV and MCH in the proband and her father, while the proband's mother showed normal hematological results. As delineated in Figure 1, the subsequent Hb capillary electrophoresis results demonstrated a decreased level of Hb A2 (2.3%), an increased level of Hb F (3.8%), and an abnormal Hb band in Hb zone 6 (9.8%) in the proband. In addition, the proband's father also showed similar Hb capillary electrophoresis results. However, only a decreased level of Hb A2 (2.5%) was observed in the proband's mother.

### 3.2. Common Thalassemia Genetic Testing Results

Common thalassemia genetic testing based on the PCR-RDB technique was used to detect the 23 common *α*-thalassemia and *β*-thalassemia variants in Chinese populations in this family. However, as demonstrated in Table 1, none of the common mutations and deletions of *α*-thalassemia and *β*-thalassemia were observed. Thus, the family members were suspected to be rare or novel globin gene variant carriers that were responsible for the abnormal hematological results.

### 3.3. Long-Read Sequencing Results

**Figure 2:**
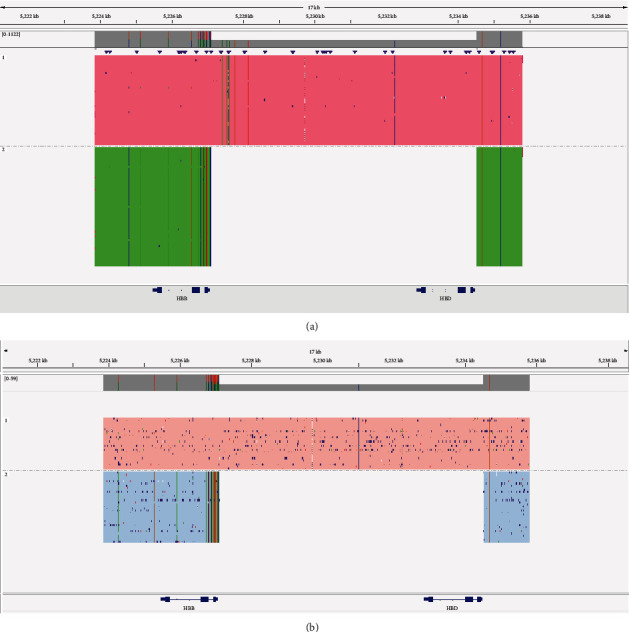
Long-read sequencing results of the enrolled family. (a) In the proband, a 7.414 kb deletion (NG_000007.3:g.63511_70924del) that partially covered *HBB* and *HBD* globin genes was identified using long-read sequencing. (b) Parental long-read sequencing detection indicated that the novel deletion in the proband was inherited from the father.

In order to further reveal the possible globin gene variants in the family, long-read sequencing was performed for globin gene sequencing including single nucleotide variation and structure variants. As shown in Figure 2, a large novel 7.414 kb deletion (NG_000007.3:g.63511_70924del) that partially cover*HBB* and *HBD* globin genes causing delta-beta fusion gene was identified by long-read sequencing in the proband. Further, parental long-read sequencing results demonstrated the same delta-beta fusion gene in the proband's father. In this family, none of the globin gene variants were observed in the proband's mother. In this family, no additional members were available for further genetic investigation.

### 3.4. Specific Gap-PCR Amplification Confirmed the Novel Deletion

**Figure 3:**
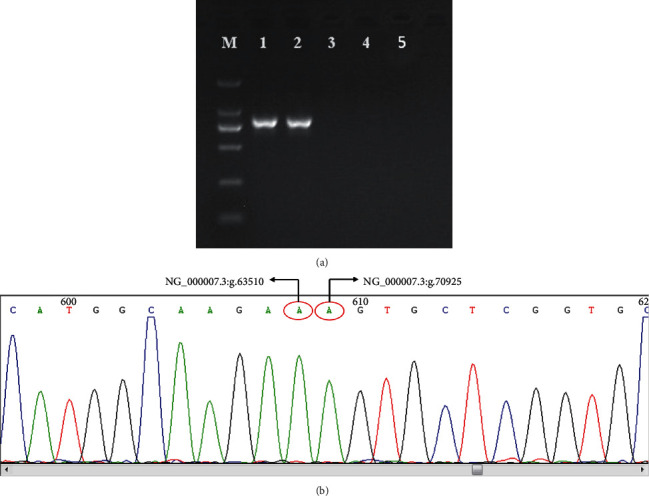
Specific gap-PCR amplification and Sanger sequencing results in the family. (a) (1) Proband. (2) Proband's father. (3) Proband's mother. (4) Normal control. (5) Blank control. (b) Comparison between sequencing results and NG_000007.3 sequence through BLAST analysis showed that the specific deletion fragments of fracture was range from 63511 to 70924 bp.

In order to verify the *HBB* and *HBD* deletions in the proband, specific gap-PCR amplification was subsequently performed. We designed the specific primers and amplified the new breakpoint in the gap-PCR technique. The gap-PCR detection results elicited a large deletion covering the *HBB* and *HBD* globin gene clusters in the proband and the proband's father. As shown in Figure 3, electrophoretic results showed that the primers P1 and P2 combination amplified a 0.8 kb PCR product in the proband and the proband's father (Figure 3).

### 3.5. Sanger Sequencing of Specific Products Amplified by Gap-PCR

In addition, in order to verify and confirm the specific location of the breakpoint, the specific products of this family amplified by gap-PCR were subsequently subjected to Sanger sequencing. Comparison between sequencing results and the NG_000007.3 sequence through BLAST analysis showed that the novel partial *HBB* and *HBD* deletion fragments of fracture were ranged from 63511 to 70924 bp, and the lack of the fragment length was 7.414 kb (NG_000007.3:g.63511_70924del) (Figure 3), which was consistent with the long-read sequencing result. Finally, we named the novel Hb Lepore variant as Hb Lepore-Quanzhou.

## 4. Discussion

The prevalence of thalassemia is high in southern China. Despite the traditional prevention and control of thalassemia carried out in South China, there are still many children born with intermediate or severe thalassemia every year. At present, blood routine analysis, Hb electrophoresis analysis, and common thalassemia gene testing based on PCR-RDB technology were used as traditional technologies to prevent and control thalassemia [22, 23]. However, there are still some limitations in the traditional technologies. Some individuals with silent thalassemia and minor thalassemia may be missed diagnosed. In addition, rare and novel globin gene variants cannot be diagnosed using traditional technologies. The detection of globin gene variants based on next-generation sequencing can effectively detect sequence variants [24, 25], while some rare structural variations may also be missed diagnosed. Thalassemia detection based on long-read sequencing technology has significant advantages in thalassemia molecular diagnosis [15, 17–20]. In this study, long-read sequencing was performed to investigate globin gene variants in a Chinese family with abnormal hematological screening results. A novel large 7.414 kb deletion NG_000007.3:g.63511_70924del partially cover *HBB* and *HBD* globin genes causing delta-beta fusion gene was identified in this family.

At present, over 300 *β*-globin gene variants have been identified. Among them, 129 genotypes have been found in the Chinese population and 16 kinds of deletional *β*-thalassemia [26]. Deletional *δβ*-thalassemia is rare in the Chinese population; among them, the Chinese Taiwan type, ^G^*γ*^+^(^A^*γδβ*)^0^, SEA-HPFH structural variants were relatively common [27]. The Lepore hemoglobins are a group of rare structural defects resulting from different recombination events between the *δ*- and *β*-globin genes, resulting in *δ* and *β* fusion genes and finally leading to *δβ*-thalassemia. Hb Lepore Boston-Washington, Baltimore, Hollandia, and Leidan have been regularly described in the database and literature [28–32]. Hb Lepore occurs worldwide, but it seems more frequently in Southern Europeans [33] and is rarely observed in Chinese populations. Despite that, Hb Lepore-Boston-Washington was indicated to be the most prevalent Hb Lepore variant in the Chinese population [34].

Presently, more and more novel Hb Lepore variants have been identified, and the average values of hematological findings in different Hb Lepore heterozygotes are listed in Table 2. A previous study conducted by Jiang et al. [35] identified a new *δ* and *β* fusion gene (NG_000007.3:g.63154_70565del), causing *δβ*-thalassemia in a Chinese individual named Hb Lepore-Hong Kong. In addition, a novel Lepore variant named Hb Lepore-ARUP (*δ*31Leu/*β*51Thr) was identified in Utah, USA [36]. Homozygous or compound heterozygosity for Hb Lepore would lead to *β*-thalassemia intermedia or major [37]. Therefore, it is of great significance to identify hemoglobin Lepore carriers during pregnancy or prenatal care to prevent the birth of *β*-thalassemia intermedia or major.

A previous study indicated that individuals with Hb Lepore would exhibit Hb Lepore bands ranging from 6 to 15% with normal or reduced Hb A2 and increased Hb F levels [38]. As delineated in Table 2, Hb Lepore might be confused with a high Hb A2 using high-performance liquid chromatography (HPLC); a range of 10-15% Hb A2 levels should be regarded as a possible Hb Lepore carrier [39]. Despite that Hb Lepore can be presumptively identified by Hb electrophoresis or reversed-phase HPLC [33], interestingly, a previous study demonstrated a novel Hb Lepore-Hong Kong without an Hb Lepore band using Hb capillary electrophoresis analysis, which was inconsistent with other Hb Lepore variants [35]. It is known that the *βδ* fusion gene is inefficiently expressed due to its promoter belonging to the *δ*-globin gene, which expresses only about 3.0% compared to the *β*-globin gene [35, 40]. In the novel Hb Lepore-Hong Kong, no Hb Lepore band was observed, which may be due to the destruction of the promoter in the *δ*-globin gene. However, consistent with other Hb Lepore variants, the individuals in our study carrying the new Hb Lepore variant exhibit amicrocytic hypochromia and an abnormal Hb Lepore band using Hb electrophoresis, which indicates the new Hb Lepore variant may also lead to *δβ*-thalassemia. In this study, none of the other hematological screening was performed such as HPLC.

In conclusion, for the first time, we described a large novel 7.414 kb deletion NG_000007.3:g.63511_70924del partially cover *HBB* and *HBD* globin genes in a Chinese family, which may cause *δβ*-thalassemia. In addition, this new Hb Lepore variant type was named Hb Lepore-Quanzhou. This finding may enrich the mutant spectrum of Hb Lepore variants and further enhance the applied advantages of long-read sequencing in the molecular diagnosis of rare and novel thalassemia.

## Figures and Tables

**Table 1:** The hematological screening and molecular analysis results of this family.

Parameters	Proband	Proband's father	Proband's mother
Sex-age	F-4	M-33	F-31
RBC (10^12^/L)	5.25	6.31	4.16
Hb (g/L)	105	139	121
MCV fL)	62.5	70.7	89.4
MCH (pg)	20	22	29.1
Hb A (%)	84.1	86.3	97.5
Hb A2 (%)	2.3	2.4	2.5
Hb F (%)	3.8	1.0	0
Hb zone 6 (%)	9.8	10.3	0
Thalassemia genotype	Hb Lepore-Quanzhou	Hb Lepore-Quanzhou	Normal

M: male; F: female; N: normal; Hb: hemoglobin; MCV: mean corpuscular volume; MCH: mean corpuscular hemoglobin.

**Table 2:** Comparing the average values of hematological findings in different Hb Lepore variants.

Lepore Hbs	Leiden [30]	Hong Kong [34]	Boston-Washington [36]	Hollandia [36]	Baltimore [36]	ARUP [36]	Quanzhou (our study)
Sample numbers	1	3	46	9	2	1	2
RBC (10^12^/L)	6.10	5.07	5.87	6.09	6.34	6.07	5.78
Hb (g/L)	142.00	117.00	131.00	132.00	143.00	120.00	122.00
MCV (fL)	71.00	71.97	77.20	69.50	77.40	70.00	66.60
MCH (pg)	22.90	22.83	24.00	21.80	22.60	19.80	21.00
Hb A2 (%)	12.10	2.43	13.10	12.00	12.40	10.30	2.35
Hb F (%)	5.50	6.60	4.09	2.84	3.05	2.00	2.40
Hb zone 6 (%)	/	0	/	/	/	/	10.05

Hb: hemoglobin; MCV: mean corpuscular volume; MCH: mean corpuscular hemoglobin.

## Data Availability

The datasets used and analyzed in the current study were obtained from the corresponding author on reasonable request.
